# The King Edward VII. Sanatorium Report

**Published:** 1914-05-02

**Authors:** 


### IS TUBERCULIN ANY USE?

### The King Edward VII. Sanatorium Report.

BY A TUBERCULOSIS MEDICAL OFFICER.

A VEXED question in all conscience is the value of tuberculin as a therapeutic measure in the treatment of tuberculous disease in general, and of phthisis in particular. Nothing approaching a definite conclusion has yet been pronounced by any of the large band of workers in this country or in Germany. True that several authorities have committed themselves to favourable statements based upon their own results; but these, as any trained statistician would at once perceive, are not accompanied by the requisite data to enable an impartial verdict to be given. Equally good results have been claimed without the use of tuberculin, and it is undeniable that, even in careful hands, the harmlessness of tuberculin is open to question.

Owing to the impetus which has been given of late years to the campaign against tuberculosis, particularly as regards the wholesale treatment of consumption, the question of the place to be assigned to tuberculin in therapeutics is one which very closely concerns the whole medical profession, above all the general practitioner and the tuberculosis officer.

The technique of tuberculin administration is exacting and cumbersome, and in dispensary practice makes great inroads on the time of the medical officer; but that would be of small account could it be shown that definite results are being obtained. It is the growing feeling that after all the treatment is of little real use which is so discouraging. To spend eighteen months in making a patient tolerant to large doses of tuberculin, and then to find that this acquired property is of doubtful value, is poor compensation; during the process other patients similarly treated may not have responded, and more than likely a few may have been definitely set back.

Practically all workers with tuberculin are agreed upon one point at least; that is, the very rigorous selection of cases necessary before embarking on the treatment. What this means in dispensary work in the Metropolis is only too clear; it means that a strict observance of all the unfavourable factors would eliminate nearly all the tuberculous patients of the district. Can it be wondered, then, that "cod-liver oil and malt" is still being doled out to an ever-increasing dispensary population. May we not soon have to ask

> Can she count
> These oil eaters with large live mobile mouths
> Agape . . . .*

A praiseworthy effort to shed light on the "tuberculin problem" has just been published in the form of a preliminary report on "The Treatment of Pulmonary Tuberculosis with Tuberculin," by Dr. Noel Bardswell (H. K. Lewis, 6s.). This report has been published at the request of the consulting staff of the King Edward VII. Sanatorium, at Midhurst. The details given in the report are extensive, and are well worth studying by those working with tuberculin, if only to impress upon them the number of factors which need careful consideration before any deductions can be drawn from the results of the treatment.

* *Casa Guidi Windows.*

The report is preceded by a prefatory note which is sure to excite interest, as it is by that trenchant critic of statistics, Professor Karl Pearson. His preface is of the nature of an interim report merely to show the grounds why it appeared to him unsafe to draw any conclusions from the data provided, in spite of the apparent detail. Correction is needed for a long series of modifying factors, which will mean a work of three or four months to complete. Meanwhile, he points out that the absence or presence of T.B. as a test of condition at the end of treatment can only be satisfactory if it does not refer merely to the last examination, and that due weight must be given to every sputum examination in relation to the length of time in the sanatorium, and to the length of time under tuberculin treatment.

It is obvious that a judgment of "lost T.B." would include in the same category three such diverse cases as these:—

(*a*) + + + − + − − − − −
(*b*) − + −
(*c*) + + + − + + − + −

so that the observations must be weighted as well as graduated in order that it may be determined whether the prevalence of T.B. is reduced more by tuberculin with sanatorium treatment, or by the sanatorium treatment alone. Professor Karl Pearson also requires further survival data before he will allow dogmatic statements on the point whether reduced T.B. prevalence on discharge is markedly associated with a greater survival rate after leaving the sanatorium.

The all-important factor of the very marked selection of patients for tuberculin treatment receives detailed consideration. This factor is one which, although now recognised as vitiating to a large extent all deductions from the results of tuberculin treatment, is still too often conveniently ignored. The initial states of the patients require to be equalised, and regard must be paid to age, weight for age, temperature range, etc., besides the actual Turban grouping. This method of grouping in itself has grave defects, seeing that it allows of only three groups and takes not sufficiently into account what may be called the systematic condition.

Many tuberculosis officers have pointed this out, and it has been suggested that a better classification would be one modelled on Sir R. W. Philip's method, but limited to six classes instead of twelve—namely, $L_1s$, $L_1S$, $L_2s$, $L_2S$, $L_3s$, $L_3S$; where L refers to the local mischief and s and S denote slight or great systematic disturbance.

Brief examples of the fallacies which are met with in taking "facts" at their face value are given by Professor Pearson in connection with the comparison of results obtained in similar groups by differing methods of treatment. These should form a salutary object lesson for enthusiasts. In Group I. cases it is possibly idle to consider the question of tuberculin at all, at any rate if they are undergoing sanatorium treatment, and Group III. cases can mostly be left out of account, as few claim that tuberculin produces much favourable effect in this class of case, so that the main issue chiefly concerns Group II. cases, unless, indeed, we require to consider whether tuberculin has any retarding effect on Group I. cases.

It must be remembered that this report is solely concerned with sanatorium patients, and that for this reason many factors have to be allowed for in deducing an opinion, which would not enter into the consideration of dispensary statistics; but, on the other hand, the patients here dealt with are under rigorous supervision, and their records have the advantage of uniformity.

The conclusions arrived at, as might be expected, are not final, especially as they refer only to eighteen months' work with tuberculin, but, nevertheless, the report is an exceedingly useful piece of work, as it is quite impartial in tone, seeking neither to decry nor exalt the therapeutic use of tuberculin. It is well that due attention is called to cases in which the evidence all points to the real possibility of tuberculin being directly harmful, for of late many have declared that the present or second "tuberculin era" of smaller doses is without adverse effect.

Dr. Noel Bardswell's experience gives confirmation to the current general impressions. Tuberculin is not going to revolutionise either our sanatorium results or the outlook for the consumptive patient. It will produce no striking results even in the most favourable cases. Tolerance may be produced, as is well known; but whether this tolerance is of much value to the patient is a question which is not yet answered. The local effect on the disease structures only occurs in some cases, and this may undoubtedly be a source of danger, while in many cases there is no obvious influence on the lesions. As far as the Midhurst results show, tuberculin cannot be looked upon as a means of turning the scale in a patient's favour when his progress is doubtful, and certainly not when it is retrogressive; in such cases it will probably do harm.

Tuberculin is now being more widely used owing to the opening up of so many new centres for treatment and the desire of the new generation of tuberculosis officers to "try" it for themselves. Dr. Bardswell has grave doubts whether this is in the interest of the average patient, and is emphatic on the necessity of avoiding reactions in giving tuberculin. This is a point on which there has been divergence of opinion, but it can hardly be doubted that safety lies in this course.

The statistical data in this report do not show that obvious advantage of tuberculin treatment which would raise it above all suspicion of showing such advantage because of the selection of patients. Further investigation may disclose a credit, or even a debit; for this we must wait. What is needed is an investigation on the following lines: Let a number of patients be selected as being *all* suitable for tuberculin treatment; then treat half of these with tuberculin plus sanatorium routine and the other half with simple sanatorium treatment. In three years' time some definite evidence of the value of tuberculin could be deduced from the histories of the cases.

[Note.—The writer seems unaware that this has been already done by others, *e.g.* by Turban, who put half his sanatorium on tuberculin and the other half on the usual treatment alone.]

